# Sustainable Starch-Based Films from Cereals and Tubers: A Comparative Study on Cherry Tomato Preservation

**DOI:** 10.3390/polym16202913

**Published:** 2024-10-16

**Authors:** Kelly J. Figueroa-Lopez, Ángel Villabona-Ortíz, Rodrigo Ortega-Toro

**Affiliations:** 1 Food Packaging and Shelf-Life Research Group (FP&SL), Food Engineering Department, Universidad de Cartagena, Cartagena 130015, Colombia; 2 Ethnopharmacology, Natural Products, and Food Research Group (GIEPRONAL), School of Sciences, Technology and Engineering, Universidad Nacional Abierta y a Distancia (UNAD), Bogotá 110911, Colombia; 3 Chemical Engineering Department, Universidad de Cartagena, Cartagena 130015, Colombia; avillabonao@unicartagena.edu.co

**Keywords:** amylose, food packaging, food shelf life, water permeability, mechanical properties

## Abstract

Biodegradable films are sustainable alternatives to conventional plastics, particularly in food preservation, where the barrier and mechanical properties are crucial for maintaining the physicochemical, microbiological, and sensory qualities of the product. This study evaluated films made from starches of corn, potato, cassava, yam, and wheat to determine their effectiveness in preserving cherry tomatoes. Amylose content, a key factor influencing the crystallinity and properties of the films, varied among the sources, with wheat starch having the highest (28.2%) and cassava the lowest (18.3%). The wheat starch film emerged as the best formulation, exhibiting the highest tensile strength and the lowest water vapor permeability (4.1 ± 0.3 g∙mm∙m^−2^∙h^−1^∙KPa^−1^), contributing to superior barrier performance. When applied to cherry tomatoes, the films based on wheat and corn starch showed the least moisture loss over fifteen days, highlighting their potential in fresh food preservation. These results suggest that starch-based films, specifically those rich in amylose, have significant potential as biodegradable packaging materials for food product conservation.

## 1. Introduction

Biodegradable packaging has gained significant attention as an eco-friendly alternative to conventional plastic packaging, particularly in the food industry [1]. The rise in environmental concerns and the push for sustainable development have driven the search for materials that can reduce plastic waste [2,3]. Biodegradable films, often derived from natural polymers such as starch, cellulose, and proteins, offer numerous advantages including renewability, compostability, and reduced environmental impact [4,5]. These materials are especially relevant in food packaging, where maintaining product quality while minimizing the environmental footprint is critical [6]. The development of biodegradable packaging is essential in addressing the global challenge of plastic pollution while meeting the consumer demand for sustainable products [7].

Starch, a natural polymer abundant in various plants, is one of the most promising materials for producing biodegradable films due to its wide availability and favorable film-forming properties [8]. Corn starch is commonly used because of its high amylose content, which contributes to its good film-forming ability and desirable mechanical properties [9]. Potato starch is known for its high molecular weight and low lipid content, which influences its film transparency and flexibility [10]. Cassava starch, with its low amylose content, offers films with high solubility in water, making it suitable for specific applications [11]. Yam starch, similar to potato starch, provides flexibility and moderate barrier properties due to its amylose and amylopectin balance [12]. Wheat starch, with the highest amylose content among the starches studied, typically forms strong, crystalline, and less permeable films, making it an excellent candidate for food packaging applications [13,14]. The physicochemical properties of these starches including amylose content, molecular structure, and gelatinization characteristics play a crucial role in determining the performance of the resulting films [15].

Several studies have explored the development of starch-based films for food packaging applications. For instance, Tongdeesoontorn et al. [16] developed biodegradable films from cassava starch blended with carboxymethyl cellulose and reported improved mechanical and barrier properties, making them suitable for fresh produce packaging. Mansur Tavares et al. [17] found that incorporating carboxymethyl cellulose (CMC) into corn and cassava starch films significantly enhanced their tensile strength, rupture stress, and rupture strain. Specifically, corn starch/CMC films, due to their higher amylose content, became more hydrophobic and showed a 48% decrease in water vapor permeability, making them more suitable for food packaging applications. Jiang et al. [18] developed pea starch-based films enhanced with potato starch nanoparticles (SNPs), which improved their mechanical properties, reduced water permeability, and increased thermal stability. Adding 6% SNPs increased the tensile strength from 8.8 MPa to 15.0 MPa, making the films suitable for degradable packaging. Basiak et al. [19] evaluated starch-based edible films for packaging applications, highlighting how the starch type (wheat, corn, or potato) and amylose content affected their properties. Potato starch films offered better barriers to oxygen and water vapor but were mechanically weaker. Higher amylose content increased moisture sensitivity and decreased wettability, making the films suitable for high-water-activity foods. Films with more amylose were thicker and opalescent, while those with less were thinner and transparent. Żołek-Tryznowska et al. [20] compared starch films made from maize, potato, oat, rice, and tapioca using 50% glycerol as a plasticizer. The study evaluated properties such as tensile strength, water vapor permeability, moisture content, wettability, and surface-free energy (SFE). Potato starch films exhibited the highest tensile strength, while oat and tapioca films had the lowest. Considering the reports, we can see that starch, particularly with a high amylose content, significantly enhances the properties of food packaging films. These films not only provide improved tensile strength and a better water vapor barrier, but are also well-suited for high-moisture products.

Perishable food products, such as fruits and vegetables, require effective packaging solutions to maintain their freshness and extend their shelf life [21]. The quality of these products is highly susceptible to environmental factors like humidity, oxygen, and temperature, which can lead to rapid deterioration [22]. Biodegradable films made from natural polymers like starch have shown great potential in preserving the quality of perishable foods by acting as barriers to moisture and gases, thereby reducing spoilage and extending shelf life [23]. The use of such films is not only beneficial for maintaining food quality, but also aligns with the growing demand for sustainable packaging solutions that minimize the environmental impact of food waste and plastic pollution [24].

This research explores the development and application of biodegradable films based on starches from different sources, aiming to evaluate their effectiveness in tomato cherry preservation. By comparing films made from corn, potato, cassava, yam, and wheat starches, the study sought to identify the most suitable materials for creating efficient food packaging solutions. Although there have been studies on the film and coating properties of starch from different sources, there are limited studies on determining the superior properties for coating purposes by comparatively evaluating the most common ones together.

## 2. Materials and Methods

### 2.1. Materials

Corn, potato, cassava, yam, and wheat starch were provided by the Association of Tuber Producers (San Juan Nepomuceno, Bolívar, Colombia). Glycerol, soy lecithin, and other reagents were provided by Sigma-Aldrich and Panreac (Bogotá, Colombia).

### 2.2. Determination of Amylose Content

The amylose content in cereals and tubers was assessed using a modified version of Juliano’s method [25]. A sample of 100 mg of starch was dissolved in 10 mL of 1 N NaOH solution and then gelatinized by heating at 90 °C. To neutralize the solution, 5 mL of 1 N acetic acid was gradually added. Subsequently, 1 mL of iodine solution (prepared by dissolving 0.2 g of iodine and 2 g of potassium iodide in 100 mL of distilled water) was mixed into the starch solution. The absorbance of this mixture was measured at 620 nm with a spectrophotometer and compared against a standard curve for pure amylose. The amylose concentration was then calculated using the regression equation derived from the standard curve.

### 2.3. Film Elaboration

Biodegradable films were developed using the solvent-casting method. Starches from corn (Fc), potato (Fp), cassava (Fm), yam (Fy), and wheat (Fw) were used in the process. The starch was dispersed at a concentration of 2% *w*/*w* in distilled water and heated to 90 °C in a water bath, with glycerol added at 20% *w*/*w* relative to the starch content. This percentage of glycerol was selected based on the research group’s experience with this type of material. Typically, 20% to 25% glycerol can be used relative to starch. A 20% glycerol content allows for the production of starch-based films with a good balance between elongation capacity and tensile strength. These film-forming dispersions (FFDs) were poured onto Teflon plates and dried at 60 °C for approximately 10 h. Once dried, the films were removed from the molds and conditioned in desiccators at 53% relative humidity (RH) for one week before characterization.

### 2.4. Film Characterization

#### 2.4.1. Thickness

Before testing, the thickness of the films was measured using a digital micrometer (TL268, Shanghai, China) with an accuracy of ±0.001 mm. Measurements were taken at five different points—two at each end and one in the center—and then averaged.

#### 2.4.2. Mechanical Properties

The mechanical properties were evaluated on samples that had been pre-conditioned for either 1 or 5 weeks at 25 °C and 53% relative humidity (RH). Approximately seven replicates of each sample were tested. The tensile strength (TS), elastic modulus (EM), and elongation (E) of the films were measured following the ASTM D882 standard [26]. EM was assessed using a universal testing machine (model TA.XTplus, Stable Micro Systems, Haslemere, UK), while TS and E were determined from stress–strain curves derived from the force-distance data of the films (1 cm wide and 10 cm long). Balanced samples were clamped in the testing machine’s jaws and stretched at a speed of 50 mm/min until they broke. During testing, the environmental RH was kept at around 53% and the temperature at 25°.

#### 2.4.3. Moisture Content (Xw)

The moisture content was calculated by averaging the measurements from film samples tested in triplicate. Initially, the samples were pre-conditioned at 53% relative humidity (RH). Samples were then dried for 24 h at 60 °C in a natural convection oven (J.P. Selecta, S.A., Barcelona, Spain). Following the drying process, the samples were stored in a desiccator containing P_2_O_5_ at 25 °C for two weeks.

(1) $$Xw=\left(\frac{W_{w}}{W_{T}}\right)$$

where % *Xw* is the moisture content; *Ww* is the weight of water in the sample (initial weight − final weight); and *WT* is the total weight of the dry sample (final weight).

#### 2.4.4. Solubility in Water

The water solubility of the films was assessed in triplicate for each formulation, measuring both the initial and final weights. The films were first immersed in distilled water at a 1:10 ratio (film to water) for 48 h. Following immersion, the samples were placed in a natural convection oven (J.P. Selecta, S.A., Barcelona, Spain) at 60 °C for 24 h to evaporate free water. Subsequently, samples were transferred to a desiccator with P_2_O_5_ at 25 °C for two weeks to eliminate any tightly bound water.

#### 2.4.5. Water Absorption Capacity

The water absorption capacity of the films was measured following the ASTM-D570 standard [27]. Dried films were immersed in about 20 mL of distilled water for 30 min, after which the samples were weighed again to assess the amount of water absorbed. The percentage of water absorbed was then calculated using Equation (1).

(2) $$\%\;Absorpion=\;\frac{Humid\;film\;weight-\;Dry\;film\;weight}{Dry\;film\;weight}\times 100$$

#### 2.4.6. Water Contact Angle (CAw)

The water contact angle of distilled water on the analyzed films was measured by studying the shape of a drop of water (0.01 mL) after 60 s using a digital camera with a white background. The distance was 50 cm from the camera lens to the water drop. The analysis of the images was carried out using Goniotrans Pro Software Version 1.0.

#### 2.4.7. Barrier Properties

The water vapor permeability (WVP) of the films was determined following the ASTM E96-95 standard [28]. The samples were conditioned for 1 and 5 weeks in a hermetic desiccator at 25 °C and 53% RH. The films were chosen based on the absence of physical defects. First, distilled water was incorporated into Payne permeation vessels (3.5 cm diameter, Elcometer SPRL, Hermelle/s Argenteau, Belgium) to expose the film to 100% RH on one side. Then, each cup was placed in a cabinet of balanced relative humidity at 25 °C, with a fan placed in the upper part of the cup to reduce the resistance to the transport of water vapor, avoiding the stagnant layer effect in this exposure. It should be noted that the RH of the cabinets (53%) was kept constant by using supersaturated solutions of magnesium nitrate-6-hydrate. The free surface of the film during film formation was exposed to the lowest relative humidity to simulate the actual application of films in high water activity products when stored at intermediate relative humidity. Subsequently, the cups were periodically weighed (0.0001 g). The water vapor transmission (WVTR) was determined from the slope obtained from the regression analysis of the weight loss data about time, once the state stationary, divided by the area of the film. Considering the WVTR data, the vapor pressure on the inner surface of the film (P_2_) was obtained with Equation (2) to correct the effect of the concentration gradients established in the stagnant air space inside the bowl.

(3) $$WVTR=\;\frac{{PDL}_{n}\;\left[\frac{P-P_{2}}{P-P_{1}}\right]}{RT\;∆\;z}$$

where P is the total pressure (atm); D is the diffusivity of water through the air at 25 °C (m^2^/s); R is the gas law constant (82.057 × 10^−3^ m^3^ atm kmol^−1^ K^−1^); T is the absolute temperature (K); Δ z is the mean height of the stagnant air gap (m), considering the initial and final z value; p 1 is the water vapor pressure on the surface of the solution (atm); and P_2_ is the corrected water vapor pressure at the inner surface of the film (atm). The water vapor permeability was calculated with Equation (3) as a function of P_2_ and P_3_ (pressure on the outer surface of the film in the cabinet). Permeability was achieved by multiplying the permeability by the average film thickness.

(4) $$Permeance=\;\frac{WVTR}{P_{2}-P_{3}}$$

The evaluation of oxygen permeability (OP) was carried out in triplicate with the Mocon OX-TRAN Model 2/23 ML equipment (Lippke, Neuwied, Germany) at 53% RH and 25 °C. The yam starch film samples were conditioned previously for 1 or 5 weeks at 25 °C and 53% RH using saturated solutions of magnesium nitrate-6-hydrate. Two samples were used in the equipment for the analysis, and they were conditioned in the cells for six hours. Then, the transmission values were determined every 20 min until equilibrium was reached. The exposure area during the tests was 50 cm^2^ for each formulation. It should be noted that the thickness of the film was considered in all cases to obtain oxygen permeability.

#### 2.4.8. Optical Properties

The Kubelka–Munk theory was used to establish the properties of spectral reflectance. The transparency of the film was established by applying the Kubelka–Munk theory for multiple scattering to the reflection spectra [29]. The surface reflectance spectra of the films were determined from 400 to 700 nm with a CM-3600d Spectro-Colorimeter (Minolta Co., Tokyo, Japan) on a black and white background. Considering that as light passes through the film, it is absorbed and partially scattered, the absorption (K) and scattering (S) coefficients can be quantified. The internal transmittance (Ti) of the films was quantified using Equation (4). Thus, R0 is the reflectance of the film on an ideal black background. Parameters a and b were calculated using Equations (5) and (6), where R is the reflectance of the sample layer supported by a known reflectance Rg. Measurements were taken in triplicate for each sample on the free film surface. A wavelength of 450 nm was considered for the analysis.

(5) $$Ti=\;\sqrt{{\left(a-R_{0}\right)}^{2}-b^{2}}$$

(6) $$a=\;\frac{1}{2}\;(R+\;\frac{R_{0}-R+R_{g}}{R_{O}R_{g}})$$

(7) $$b=\;\sqrt{a^{2}-1\;\;}$$

The gloss was determined according to the ASTM D523 standard [30], on the surface of the free film, with an angle of incidence of 60°, using a flat surface gloss meter (3nh Gloss Meter, Guangzhou, China). For this, measurements were taken in triplicate for each sample, and three films of each formulation were considered. All of the results were expressed as gloss units; a highly polished surface of black glass standard with an approximate value of 100. The transparency and gloss measurements were carried out with films conditioned for one week (initial) and five weeks (end time) in hermetic desiccators at 25 °C and 53% RH.

### 2.5. Film Application as a Cherry Tomato Coating

The application of the coatings on cherry tomatoes was carried out by the immersion method, according to what was reported by Rivera et al. [31]. Uncoated tomatoes were taken as negative targets. The tomatoes were selected, washed, and disinfected before coating. Twenty tomatoes were used for each formulation. The method used for coating the tomatoes was direct immersion of the tomatoes into the film-forming dispersions. The immersion was carried out for 1 min at 25 °C. Afterward, the excess liquid was allowed to drain before storage. These were then stored at 30 °C and 70% RH for two weeks, with their weight loss recorded.

### 2.6. Statistical Analysis

Data for each test were analyzed statistically. Analysis of variance (ANOVA) was used to assess significance in the difference between factors and levels. Averages were evaluated using the Fisher least significant difference (LSD) test with 95% confidence. Data were analyzed using Statgraphics Plus Version 5.0 for Windows software (Manugistics Corp., Rockville, MD, USA).

## 3. Results

### 3.1. Determination of Amylose Content

The amylose contents of the cereals and tubers are shown in Table 1. The analysis of amylose content across various starch sources revealed significant differences in their composition. Wheat starch, with the highest amylose content at 28.2%, stood out compared to other types. Corn starch, with 25.1% amylose, also showed a high amylose content. Potato starch, containing 20.2% amylose, and cassava starch, with 18.3%, had lower amylose percentages. Yam starch, at 23.1% amylose, presented a middle ground among these sources, demonstrating moderate amylose. These values were similar to those reported by Domene-Lopez et al. [32], where corn starch had 24.8% amylose, wheat starch 24.5% amylose, and potato starch 20.5% amylose. This variation in amylose content across different starches highlights the influence of the starch source on its amylose concentration, reflecting the inherent differences in starch composition and its impact on food packaging applications.

### 3.2. Mechanical Properties

**Figure 1:**
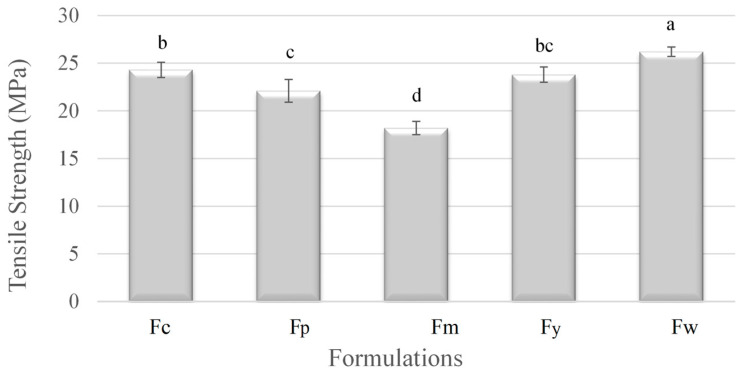
Tensile strength of samples prepared from corn (Fc), potato (Fp), cassava (Fm), yam (Fy), and wheat (Fw) starches. ^a–d^ Different superscript letters within the different columns indicate significant differences among formulations (*p* < 0.05).

Figure 1 shows the mechanical properties of the films. The tensile strength of the films varied significantly depending on the starch source, which is directly related to the amylose content. Sample Fw (wheat starch), with the highest amylose content (28.2%), exhibited the greatest tensile strength. This is because the high amylose content led to more crystalline structures, which strengthened the material. On the other hand, Fm (cassava starch), with the lowest amylose content (18.3%), had the weakest tensile strength, indicating fewer crystalline regions and a more amorphous structure. The other samples, Fc (corn starch), Fy (Yam starch), and Fp (potato starch), showed intermediate tensile strengths, reflecting their respective amylose levels. Overall, the higher amylose content in starch correlated with increased tensile strength due to enhanced crystallinity.

**Figure 2:**
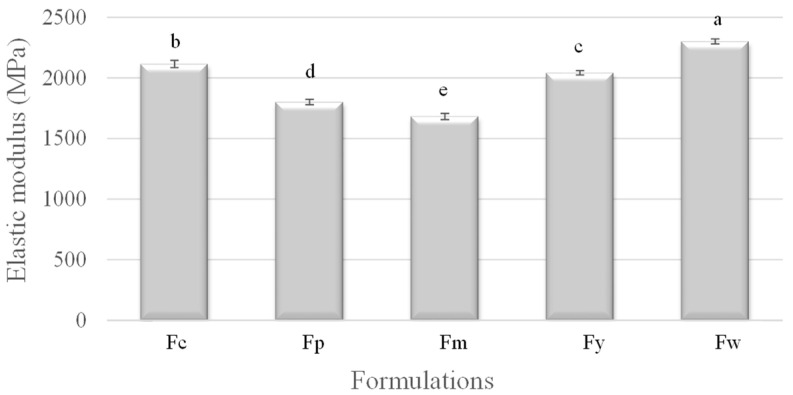
Elastic modulus of samples prepared from corn (Fc), potato (Fp), cassava (Fm), yam (Fy), and wheat (Fw) starches. ^a–e^ Different superscript letters within the different columns indicate significant differences among formulations (*p* < 0.05).

In Figure 2, the elastic modulus, which measures the stiffness of the samples, increased with amylose content. Fw (wheat starch) again exhibited the highest modulus, indicating it as the stiffest material, likely due to its high amylose content promoting strong intermolecular forces and crystalline regions. Fm (cassava starch), with the lowest amylose content, had the lowest modulus, suggesting it to be the most flexible. The other samples, Fc (corn starch), Fy (yam starch), and Fp (potato starch), fell in between, with their elastic moduli reflecting their intermediate amylose contents. This pattern shows that films with a higher amylose content are stiffer and less deformable.

**Figure 3:**
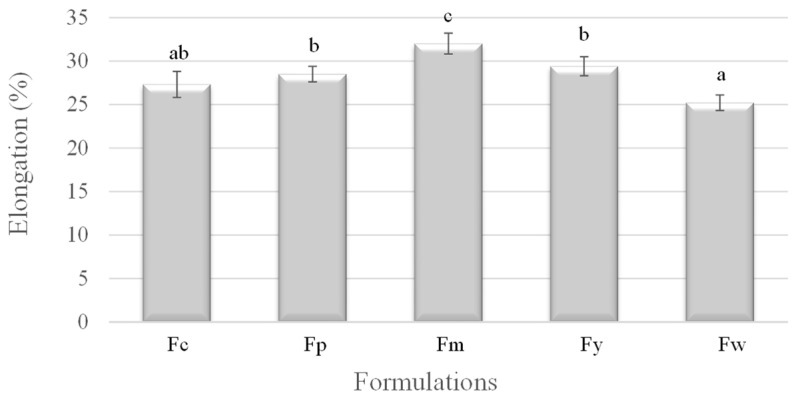
Elongation of samples prepared from corn (Fc), potato (Fp), cassava (Fm), yam (Fy), and wheat (Fw) starches. ^a–c^ Different superscript letters within the different columns indicate significant differences among formulations (*p* < 0.05).

Figure 3 illustrates the elongation at break, revealing an inverse relationship between elongation and amylose content. Fm (cassava starch), with the lowest amylose content, demonstrates the highest elongation, indicating high flexibility and ductility. In contrast, Fw (wheat starch), with the highest amylose content, had the lowest elongation, making it the most brittle. The other samples, Fc (corn starch), Fy (yam starch), and Fp (potato starch), showed intermediate elongation values, corresponding to their respective amylose contents. These results suggest that a higher amylose content leads to decreased ductility, as the material becomes more crystalline and less capable of stretching before breaking.

The mechanical properties of a starch-based film’s tensile strength, elastic modulus, and elongation are strongly influenced by the amylose content of the starch used in their formulation. A higher amylose content generally enhances the tensile strength and stiffness (elastic modulus) but reduces the film’s ductility (elongation). This analysis demonstrates the critical role of amylose in defining the mechanical behavior of starch-based films, making it a key factor in designing materials for specific applications where particular mechanical properties are desired. Similar mechanical values were reported by Domene-Lopez et al. [32], who observed the lowest tensile strength and stiffness in films with the lowest amylose percentage. Wheat and corn starch films, which had the highest amylose content, exhibited the greatest tensile strength but were less stretchable compared to the potato and rice starch films. Cano et al. [33] concluded that films rich in amylose form crystalline regions that result in stiffer, more fracture-resistant films, but with reduced stretchability. Brain Wilfer et al. [34] also reported significant changes in the mechanical strength of films made with different starches (potato, tapioca, corn, rice, and tapioca). These changes were primarily attributed to the amylose content of the samples and the use of plasticizers.

### 3.3. Moisture Content

Table 2 shows the values for the water content of the films. Water content (Xw) varied notably across the samples, with Fw (wheat starch) having the lowest water content (0.060) and Fm (cassava starch) the highest (0.090). This trend was inversely related to the amylose content; higher amylose formulations, like Fw, tend to retain less water. Amylose’s crystalline regions are less hydrophilic than amylopectin’s amorphous regions, leading to lower water retention. Consequently, films with a higher amylose content exhibit lower water content, which can enhance their stability and reduce their susceptibility to degradation in moist environments. These findings are consistent with those reported by Domene-Lopez et al. [32], who observed a low moisture content in films made from starches with high amylose content such as wheat and corn starch.

### 3.4. Solubility in Water

Table 2 presents the values of solubility in water (Sw) of the films, which also showed variation, with Fw (wheat starch) having the lowest solubility (0.25) and Fm (cassava starch) the highest (0.36). A higher amylose content in starch leads to more crystalline structures, which are less soluble in water. Thus, the lower solubility of Fw can be attributed to its higher amylose content, which forms strong, water-resistant crystalline regions, as described by Jimenez et al. [8] in their review of starch-based films. On the other hand, the lower amylose content in Fm resulted in a more amorphous structure that is more readily soluble in water, explaining its higher Sw value.

### 3.5. Water Absorption Capacity

Table 2 shows the water absorption capacity (Aw) of the films. The Aw was highest for Fm (cassava starch) at 0.86 and lowest for Fw (wheat starch) at 0.73. This trend is consistent with the amylose content, where higher amylose films absorb less water. Amylose’s crystalline regions are less capable of absorbing water compared to the amorphous regions associated with amylopectin, as was observed by Krolikowska et al. [35] in their study about cassava and wheat starches. As a result, Fw’s high amylose content made it less absorbent, while the low amylose content in Fm allowed for greater water absorption, enhancing its swelling capacity.

### 3.6. Water Contact Angle (CAw)

Table 2 shows the contact angle values of the films. The CAw is an indicator of hydrophobicity, where a higher angle suggests greater water resistance. Fw (wheat starch) showed the highest contact angle (58.5°), indicating the greatest hydrophobicity, while Fm (cassava starch) had the lowest (51.1°), indicating more hydrophilic properties. This pattern reflects the influence of amylose content; higher amylose formulations like Fw are more crystalline and therefore more resistant to water, increasing the contact angle. In contrast, the more amorphous structure of Fm, due to a lower amylose content, resulted in a lower contact angle, reflecting a higher water affinity. Basiak et al. [19] also reported the highest water contact angle values for wheat starch films, while the contact angles for corn and potato starch films were lower due to their more hydrophilic surfaces.

### 3.7. Barrier Properties

Table 3 shows the water vapor permeability (WVP) and oxygen permeability (OP) of the films. The WVP of the starch films varied significantly, with values ranging from 4.1 g∙mm∙m^−2^∙h^−1^∙KPa^−1^ for Fw (wheat starch) to 6.1 g∙mm∙m^−2^∙h^−1^∙KPa^−1^ for Fm (cassava starch). Films with higher WVP are more permeable to water vapor, which is generally undesirable for packaging applications that require strong moisture barrier properties [36]. The data revealed that films with higher amylose content, such as Fw (28.2% amylose), had lower WVP, while Fm, with the lowest amylose content (18.3%), exhibited the highest WVP. The lower WVP observed in high-amylose films can be attributed to the chemical structure of amylose, a linear polymer of glucose that tends to form tightly packed, crystalline regions within the starch matrix [37]. These crystalline regions are less permeable to water vapor because they create a dense network that reduces the diffusion pathways available for water molecules to pass through the film [38]. The strong intermolecular hydrogen bonds that form within these crystalline structures further enhance the barrier properties by limiting the free volume, making it more difficult for water vapor to penetrate the film. In contrast, amylopectin, the branched counterpart of amylose, forms a more amorphous and less ordered structure within the film. This amorphous structure, which is predominant in low-amylose formulations like Fm, has a greater free volume and a more open network. As a result, water vapor molecules can diffuse more easily through these films, leading to higher WVP values. Thus, the higher WVP in low-amylose films like Fm reflects the less compact, more permeable nature of their structure, which is less effective at blocking moisture transmission. Overall, the WVP results strongly correlate with the amylose content, demonstrating that a higher amylose content enhances the moisture barrier properties of starch films by promoting the formation of a more crystalline, impermeable matrix. Other authors have reported an increase in permeability in samples made from starches with high amylose contents, which has mainly been related to the concentration of plasticizers, inducing an increase in permeability. For instance, Bertuzzi et al. [39] reported high water vapor permeability for corn starch samples with high amylose content, while cassava starch-based films presented the lowest water vapor permeability values.

The oxygen permeability (OP) of the starch films ranged from 0.61 × 10^13^ cm^3^∙m^−1^∙s^−1^∙Pa^−1^ for Fw (wheat starch) to 0.92 × 10^13^ cm^3^∙m^−1^∙s^−1^∙Pa^−1^ for Fm (cassava starch). Lower OP values indicate better oxygen barrier properties, which are crucial for packaging materials that need to protect against oxidation [40]. The data showed that films with a higher amylose content, such as Fw (28.2% amylose), exhibited a lower OP, whereas films with a lower amylose content, like Fm (18.3% amylose), had a higher OP. The lower OP observed in high-amylose films can be explained by the chemical structure of amylose. Amylose is a linear polymer that forms tightly packed, crystalline regions within the starch matrix, creating a dense structure with fewer and more tortuous pathways for oxygen molecules to diffuse through. The compact arrangement of glucose units in amylose, coupled with strong intermolecular hydrogen bonding, restricts the movement of oxygen molecules, thereby reducing the film’s oxygen permeability. In contrast, amylopectin, which is more prevalent in low-amylose films like Fm, forms a branched, amorphous structure that lacks the tight packing found in high-amylose films. This amorphous network provides greater free volume and less resistance to the movement of oxygen molecules, leading to higher OP values. The less ordered and more open structure of low-amylose films results in a less effective oxygen barrier, allowing oxygen to diffuse more readily through the material. Therefore, the OP results correlate with the amylose content, demonstrating that a higher amylose content significantly enhances the oxygen barrier properties of starch films by promoting a more crystalline and compact structure that effectively limits oxygen diffusion.

### 3.8. Optical Properties

In Table 4 are gathered the gloss at 60° (GU) values and the internal transmittance (Ti) of the films. Gloss is a measure of how light reflects off the surface of the film, with higher values indicating a smoother and more reflective surface [41]. GU values ranged from 22.2 GU for Fw (wheat starch) to 24.0 GU for Fy (yam starch). The variation in gloss among the samples can be linked to the amylose content and the resulting microstructure of the films. Films with lower amylose content, like Fm (cassava starch), tended to have higher gloss values. This is because amylopectin, which was more abundant in these films, contributes to a more amorphous and smoother surface, allowing for greater light reflection. In contrast, films with higher amylose content, such as Fw, exhibited lower gloss values. The linear structure of amylose promotes the formation of crystalline regions within the film, which can lead to a rougher surface at the microscopic level, scattering light and reducing gloss [42]. Therefore, the lower gloss observed in high-amylose films can be attributed to the increased surface roughness caused by the crystalline regions formed by amylose, which disrupts the uniformity of light reflection [43].

The internal transmittance (Ti) at 450 nm of the films ranged from 82.0% for Fw (wheat starch) to 89.3% for Fm (cassava starch). Internal transmittance measures the ability of light to pass through the material, with higher values indicating greater transparency. The results showed that films with a higher amylose content, like Fw, had lower transmittance, while those with a lower amylose content, like Fm, exhibited higher transmittance. This trend is consistent with the structural characteristics of amylose and amylopectin. The crystalline regions formed by amylose in high-amylose films scatter light, reducing the film’s transparency [44]. These crystalline domains create a more heterogeneous internal structure, which interferes with the passage of light through the film [45]. On the other hand, the amorphous structure associated with high amylopectin content in low-amylose films allows light to pass through with less scattering, resulting in higher transmittance. Therefore, the lower internal transmittance observed in high-amylose films can be attributed to the increased light scattering caused by the crystalline regions within the film, which reduces its overall transparency [46].

It is worth noting that the optical properties are strongly influenced by the starch source (amylose/amylopectin ratio) and the thickness of the films. Samples with a higher amylose content tend to be thicker, leading to increased opacity [47]. Basiak et al. [19] obtained similar results, where potato starch-based films showed higher transparency, while corn and wheat starch films showed higher opalescence. This was mainly attributed to the amylose content and the thickness of the films. It was similarly concluded that cassava starch-based films exhibited greater transparency compared to those made from corn and yam starch. This increased transparency was attributed to the higher amylopectin content and reduced thickness of the cassava starch films. Żołek-Tryznowska et al. [20] obtained lower transparency for potato starch-based films compared to those made from tapioca, corn, or rice. Mali et al. [47] similarly concluded that cassava starch-based films exhibited greater transparency compared to those made from corn and yam starch. This increased transparency was attributed to the higher amylopectin content and reduced thickness of the cassava starch films.

### 3.9. Film Application as a Cherry Tomato Coating

**Figure 4:**
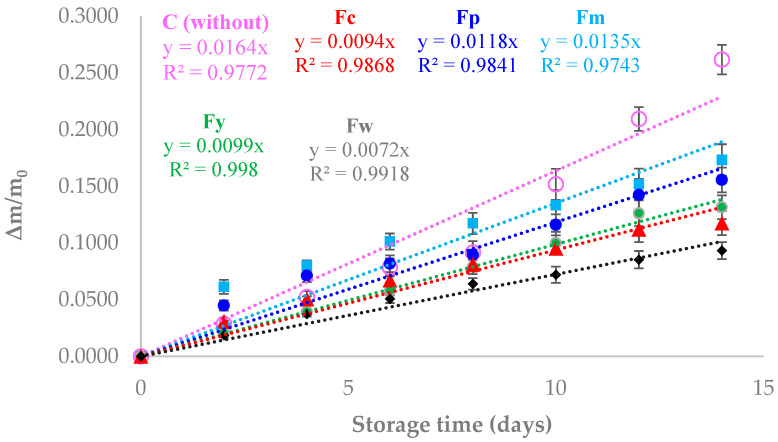
Weight loss of cherry tomatoes with and without a coating stored at 70% RH and 30 °C for two weeks.

Figure 4 illustrates the weight loss of cherry tomatoes stored under 70% relative humidity at 30 °C for two weeks, comparing those coated with different starch films to uncoated tomatoes (control). The results showed that the tomatoes coated with starch films experienced significantly less weight loss compared to the control, indicating the effectiveness of these coatings in reducing moisture loss, which is critical for maintaining food quality and extending shelf life. Tomatoes without any coating (control) showed the highest weight loss, which can be attributed to the lack of a barrier preventing water evaporation. In contrast, tomatoes coated with the different starch films exhibited reduced weight loss, demonstrating that the films acted as a moisture barrier, slowing down the rate of water evaporation. This reduction in moisture loss is crucial, as it helps maintain the firmness, texture, and overall freshness of the tomatoes, directly impacting their marketability and consumer acceptance. Among the coated samples, those with higher amylose content, such as the Fw (wheat starch) film, showed the least weight loss. This can be explained by the denser and more crystalline structure of high-amylose films, which effectively restricts the passage of water vapor, thereby better preserving the moisture content of the tomatoes. On the other hand, samples with a lower amylose content, like Fm (cassava starch), displayed a slightly higher weight loss compared to Fw, but was still significantly lower than the control. The more amorphous structure of the low-amylose films, while still providing a barrier, was less effective at preventing moisture loss compared to the crystalline structure of the high-amylose films. Overall, the results indicate that starch films, particularly those with higher amylose content, are effective in reducing the weight loss of cherry tomatoes during storage by acting as a barrier to moisture loss. This suggests that such films could be beneficial as edible coatings to extend the shelf life of fresh produce by minimizing dehydration, thereby preserving food quality over time. Other authors have reported the same trend in different food products. In this regard, Thakur et al. [48] applied a coating of rice starch and carrageenan to bananas, achieving a 40% extension to their shelf life. They also reported a delay in ethylene production and the starch degradation rate during storage. Fakhouri et al. [49] reported a reduction in weight loss and an improved appearance after 21 days of refrigerated storage of Red Crimson grapes coated with starch/gelatin-based edible films. Pellá et al. [50] succeeded in extending the shelf life of guavas by up to 2 days with a coating based on cassava starch, gelatin, and casein. They attributed the increased shelf life to the low water vapor transmission rate of the films, which led to a reduction in the fruit’s mass loss. Similarly, Gómez-Contreras et al. [51] reported a reduction in weight loss in strawberries coated with yam starch-based films and essential oils stored at 25 °C and 85% RH for two weeks.

## 4. Discussion

The results of this study provide valuable insights into the potential of starch-based films as effective coatings to preserve the quality and extend the shelf life of cherry tomatoes. The analysis of various properties including water vapor permeability (WVP), oxygen permeability (OP), gloss, internal transmittance, and the application of these films on tomatoes supports the working hypothesis that starch films, particularly those with higher amylose content, offer superior barrier properties, which are critical for food preservation. The findings are consistent with previous studies that have highlighted the role of amylose in enhancing the barrier properties of biopolymer films. High-amylose films, due to their crystalline structure, have been shown to exhibit lower WVP and OP, which directly translates into better moisture and oxygen barrier properties. This is crucial for reducing water loss and limiting oxidation in food products, both of which are primary factors affecting food quality and shelf life. The lower gloss and internal transmittance observed in high-amylose films further support the notion that these films are denser and less permeable, as the crystalline regions scatter light and reduce film transparency. These properties are desirable for packaging applications where the prevention of spoilage is more critical than the esthetic appearance of the packaging.

When applied as a coating on cherry tomatoes, the starch films demonstrated a significant reduction in weight loss compared to uncoated tomatoes, with the most substantial effect observed in films with a higher amylose content. This finding is crucial because weight loss in fresh produce is directly associated with moisture loss, leading to decreased firmness, shriveling, and overall quality degradation. The ability of these films to minimize moisture loss suggests that they can effectively slow down the ripening and spoilage processes, thereby extending the shelf life of fresh produce. This aligns with previous research indicating that edible coatings can act as semi-permeable barriers, reducing respiration rates, delaying ripening, and maintaining post-harvest quality.

The implications of these findings are significant in the context of developing sustainable and effective packaging solutions for the food industry. The use of starch-based films as edible coatings not only offers a biodegradable alternative to synthetic packaging materials, but also enhances the preservation of fresh produce, potentially reducing food waste. However, while the study demonstrates the effectiveness of these films, further research is needed to optimize their formulation and application. Future studies could explore the incorporation of natural antimicrobial agents or plasticizers to further improve the mechanical properties and functional performance of the films.

On the other hand, considering the formulations used and the properties of starch, it can be inferred that the materials obtained may be susceptible to attack by microorganisms, since starch is usually a source of energy for some bacteria and fungi. However, when stored in conditions of medium relative humidity (around 50%), they can be preserved without problems. As for their resistance to temperature changes, starch-based materials have thermoplastic behavior; if the temperature is raised above their softening temperature, these materials can be molded again at new temperatures, and above the degradation temperature, they suffer structural damage.

As can be seen from the properties of the films studied, starch-based materials were obtained in a simple way. Their effectiveness in preserving tomatoes was demonstrated, and they can also be used for preserving other food products such as fruits and vegetables. These materials are biodegradable by nature, and there has been a growth in the development of starch-based materials on a large scale, providing new development opportunities and reducing costs as industries produce them on a larger scale.

In conclusion, this study reinforces the potential of starch-based films, particularly those with higher amylose content, as a viable solution for extending the shelf life of fresh produce. The results contribute to the broader understanding of how biopolymer films can be tailored to meet specific packaging needs, supporting the ongoing shift toward more sustainable and environmentally friendly packaging technologies.

## Figures and Tables

**Table 1:** Amylose content of cereals and tubers used to obtain films.

Source	Amylose Content (%)
Corn starch	25.1 ± 0.3 ^d^
Potato starch	20.2 ± 0.4 ^b^
Cassava starch	18.3 ± 0.2 ^a^
Yam starch	23.1 ± 0.2 ^c^
Wheat starch	28.2 ± 0.3 ^e^

^a–e^ Different superscript letters within the same column indicate significant differences among formulations (*p* < 0.05).

**Table 2:** Moisture content (Xw), solubility in water (Sw), water absorption capacity (Aw), and contact angle with water (CAw) of samples prepared from corn (Fc), potato (Fp), cassava (Fm), yam (Fy), and wheat (Fw) starches.

Formulations	Thickness	Xw	Sw	Aw	CAw
Fc	185 ± 5 ^a^	0.070 ± 0.005 ^c^	0.28 ± 0.02 ^bc^	0.75 ± 0.02 ^c^	57.8 ± 0.5 ^d^
Fp	188 ± 8 ^a^	0.075 ± 0.002 ^c^	0.34 ± 0.02 ^ab^	0.83 ± 0.03 ^ab^	54.2 ± 0.5 ^b^
Fm	190 ± 6 ^a^	0.090 ± 0.003 ^a^	0.36 ± 0.02 ^a^	0.86 ± 0.02 ^a^	51.1 ± 0.3 ^a^
Fy	185 ± 4 ^a^	0.081 ± 0.003 ^b^	0.31 ± 0.03 ^b^	0.8 ± 0.03 ^b^	56.0 ± 0.5 ^c^
Fw	192 ± 5 ^a^	0.060 ± 0.002 ^d^	0.25 ± 0.02 ^c^	0.73 ± 0.03 ^c^	58.5 ± 0.4 ^d^

^a–d^ Different superscript letters within the same column indicate significant differences among formulations (*p* < 0.05).

**Table 3:** Comparison of the water vapor permeability (WVP) and oxygen permeability (OP) of samples prepared from corn (Fc), potato (Fp), cassava (Fm), yam (Fy), and wheat (Fw) starches.

Formulations	WVP × 10 (g∙mm∙m^−2^∙h^−1^∙KPa^−1^)	OP × 10^13^ (cm^3^∙m^−1^∙s^−1^∙Pa^−1^)
Fc	4.4 ± 0.2 ^c^	0.65 ± 0.02 ^c^
Fp	5.5 ± 0.4 ^ab^	0.82 ± 0.02 ^b^
Fm	6.1 ± 0.5 ^a^	0.92 ± 0.03 ^a^
Fy	5.2 ± 0.2 ^b^	0.80 ± 0.05 ^b^
Fw	4.1 ± 0.3 ^c^	0.61 ± 0.02 ^c^

^a–c^ Different superscript letters within the same column indicate significant differences among formulations (*p* < 0.05).

**Table 4:** Gloss at 60° and internal transmittance (Ti) of samples prepared from corn (Fc), potato (Fp), cassava (Fm), yam (Fy), and wheat (Fw) starches.

Formulations	Gloss at 60°	Ti at 450 (nm)
Fc	23.0 ± 0.5 ^ab^	85.6 ± 2.0 ^ab^
Fp	23.5 ± 0.3 ^a^	86.5 ± 3.0 ^ab^
Fm	23.5 ± 0.4 ^a^	89.3 ± 3.0 ^a^
Fy	24.0 ± 0.6 ^a^	86.0 ± 2.0 ^a^
Fw	22.2 ± 0.5 ^b^	82.0 ± 2.0 ^b^

^a,b^ Different superscript letters within the same column indicate significant differences among formulations (*p* < 0.05).

## Data Availability

The original contributions presented in the study are included in the article, further inquiries can be directed to the corresponding author.
